# *N*-[(*E*)-(5-Methyl­thio­phen-2-yl)methyl­idene]-1*H*-1,2,4-triazol-3-amine

**DOI:** 10.1107/S1600536808041494

**Published:** 2008-12-13

**Authors:** Zahid H. Chohan, Muhammad Hanif, M. Nawaz Tahir

**Affiliations:** a Department of Chemistry, Bahauddin Zakariya University, Multan-60800, Pakistan; b Department of Physics, University of Sargodha, Sargodha, Pakistan

## Abstract

In the title Schiff base, C_8_H_8_N_4_S, a condensation product of 5-methyl­thio­phene-2-carboxaldehyde and 3-amino-1,2,4-triazole, the dihedral angle between the triazolyl and thienyl rings is 6.44 (14)°. The compound exists as a polymeric chain arising from inter­molecular N—H⋯N bonding.

## Related literature

For a related comound, see: Chohan *et al.* (2009 ▶). For the biological properties of such compounds, see: Foroumadi *et al.* (2003 ▶); Manfredini *et al.* (2000 ▶).
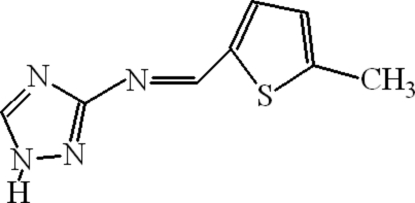


## Experimental

### 

#### Crystal data

C_8_H_8_N_4_S
*M* *_r_* = 192.24
Orthorhombic, 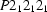

*a* = 7.2570 (7) Å
*b* = 8.9522 (8) Å
*c* = 14.2930 (15) Å
*V* = 928.56 (16) Å^3^
*Z* = 4
Mo *K*α radiation
μ = 0.31 mm^−1^
*T* = 296 (2) K
0.24 × 0.16 × 0.14 mm

#### Data collection

Bruker KAPPA APEXII CCD diffractometer
Absorption correction: multi-scan (*SADABS*; Bruker, 2005 ▶) *T*_min_ = 0.928, *T*_max_ = 0.956
5793 measured reflections
2206 independent reflections
1859 reflections with *I* > 2σ(*I*)
*R*_int_ = 0.034

#### Refinement

*R*[*F* ^2^ > 2σ(*F* ^2^)] = 0.034
*wR*(*F* ^2^) = 0.097
*S* = 1.05
2206 reflections
134 parameters
H atoms treated by a mixture of independent and constrained refinement
Δρ_max_ = 0.20 e Å^−3^
Δρ_min_ = −0.23 e Å^−3^
Absolute structure: Flack (1983 ▶), 854 Friedel pairs
Flack parameter: −0.02 (10)

### 

Data collection: *APEX2* (Bruker, 2007 ▶); cell refinement: *SAINT* (Bruker, 2007 ▶); data reduction: *SAINT*; program(s) used to solve structure: *SHELXS97* (Sheldrick, 2008 ▶); program(s) used to refine structure: *SHELXL97* (Sheldrick, 2008 ▶); molecular graphics: *ORTEP-3 for Windows* (Farrugia, 1997 ▶) and *PLATON* (Spek, 2003 ▶); software used to prepare material for publication: *WinGX* publication routines (Farrugia, 1999 ▶) and *PLATON*.

## Supplementary Material

Crystal structure: contains datablocks text, I. DOI: 10.1107/S1600536808041494/ng2524sup1.cif

Structure factors: contains datablocks I. DOI: 10.1107/S1600536808041494/ng2524Isup2.hkl

Additional supplementary materials:  crystallographic information; 3D view; checkCIF report

## Figures and Tables

**Table 1:** Hydrogen-bond geometry (Å, °)

*D*—H⋯*A*	*D*—H	H⋯*A*	*D*⋯*A*	*D*—H⋯*A*
N3—H3n⋯N1^i^	0.85 (3)	2.12 (3)	2.963 (2)	172 (2)

Symmetry code: (i) 
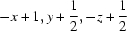
.

**Fig. 1.**
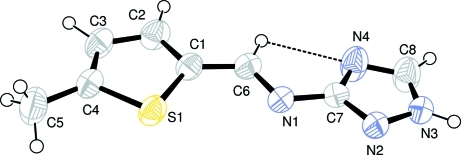
ORTEP-3 for Windows (Farrugia, 1997) drawing of the title compound, C8H8N4S, with the atom numbering scheme. The thermal ellipsoids are drawn at the 50% probability level. H-atoms are shown by small circles of arbitrary radii. The intramolecular H-bonding is shown by dashed lines.

**Fig. 2.**
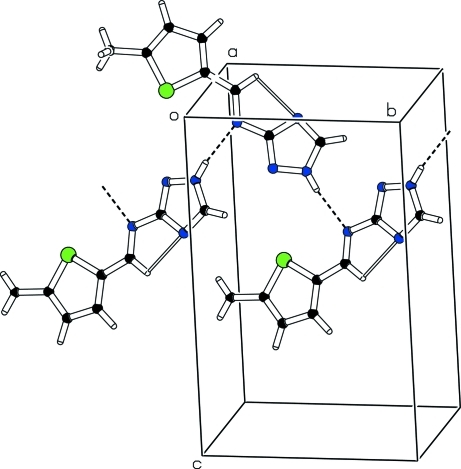
The partial unit cell packing of (I) (Spek, 2003) showing the interamolecular and intermolecular hydrogen bonding showing that polymeric sheets are formed.

## supplementary crystallographic information

### Comment

Compounds derived from triazole possess antimicrobial, analgesic, anti-inflammatory, local anesthetic, antineoplastic and antimalarial properties (Foroumadi *et al.*, 2003). Some triazole Schiff bases also exhibited antiproliferative and anticancer activity (Manfredini *et al.*, 2000). Due to their significant biological applications they have gained much attention in bioinorganic and metal-based drug discovery. In view of its structural and biological importance, we have synthesized (Chohan *et al.*, 2009), series of triazole derived Schiff bases along with the title compound (I). We report herein, its preperation and crystal structure.

In the molecule of title compound, (Fig 1), the bond lengths and angles are within normal ranges. In this molecule 5-methylthiophen ring is attached to 5-membered ring of triazole moiety through the Schiff bond C=N. The dihedral angle between ring A(S1/C1—C4) and B(C7/N2/N3/C8/N4) is 6.44 (14)°. There exist intramolecular as well as an intermolecular H-bonds as given in Table 1. The molecules are connected to each other through intermolecular H-bonds of N–H···N type in a helical way (Fig 2).

### Experimental

A mixture of 5-methylthiophene-2-carboxaldehyde (1.09 ml, 0.01 *M*) and 3-amino-1,2,4-triazole (0.84 g, 0.01 *M*) in 1:1 molar proportions in methanol (40 ml) was boiled under reflux for 5 h by monitoring through TLC. The reaction mixture was cooled at room temperature and filtered; within an hour a light brown solid product separated from the clear solution. It was filtered, washed with methanol, dried and recrystallized from a mixture of ethanol:methanol (1:1).

### Refinement

H-atoms were positioned geometrically, with C—H = 0.96 Å for methyl carbon of thiophene ring and constrained to ride on the parent atom. The coordinates of all other H-atoms were refined. The *U*_iso_(H) = *xU*_eq_(C, N), where *x* = 1.5 for methyl H and *x* = 1.2 for all other H atoms.

### Crystal data

**Table:**

C_8_H_8_N_4_S	*F*(000) = 400
*M**_r_* = 192.24	*D*_x_ = 1.375 Mg m^−^^3^
Orthorhombic, *P*2_1_2_1_2_1_	Mo *K*α radiation, λ = 0.71073 Å
Hall symbol: P 2ac 2ab	Cell parameters from 2206 reflections
*a* = 7.2570 (7) Å	θ = 2.7–28.3°
*b* = 8.9522 (8) Å	µ = 0.31 mm^−^^1^
*c* = 14.2930 (15) Å	*T* = 296 K
*V* = 928.56 (16) Å^3^	Prismatic, light brown
*Z* = 4	0.24 × 0.16 × 0.14 mm

### Data collection

**Table:**

Bruker KAPPA APEXII CCD diffractometer	2206 independent reflections
Radiation source: fine-focus sealed tube	1859 reflections with *I* > 2σ(*I*)
graphite	*R*_int_ = 0.034
Detector resolution: 7.4 pixels mm^-1^	θ_max_ = 28.3°, θ_min_ = 2.7°
ω scans	*h* = −9→9
Absorption correction: multi-scan (*SADABS*; Bruker, 2005)	*k* = −11→11
*T*_min_ = 0.928, *T*_max_ = 0.956	*l* = −17→19
5793 measured reflections	

### Refinement

**Table:**

Refinement on *F*^2^	Secondary atom site location: difference Fourier map
Least-squares matrix: full	Hydrogen site location: inferred from neighbouring sites
*R*[*F*^2^ > 2σ(*F*^2^)] = 0.034	H atoms treated by a mixture of independent and constrained refinement
*wR*(*F*^2^) = 0.097	*w* = 1/[σ^2^(*F*_o_^2^) + (0.0562*P*)^2^ + 0.0383*P*] where *P* = (*F*_o_^2^ + 2*F*_c_^2^)/3
*S* = 1.05	(Δ/σ)_max_ < 0.001
2206 reflections	Δρ_max_ = 0.20 e Å^−^^3^
134 parameters	Δρ_min_ = −0.22 e Å^−^^3^
0 restraints	Absolute structure: Flack (1983), 854 Friedel pairs
Primary atom site location: structure-invariant direct methods	Flack parameter: −0.02 (10)

### Special details

**Table:**

Geometry. Bond distances, angles *etc*. have been calculated using the rounded fractional coordinates. All su's are estimated from the variances of the (full) variance-covariance matrix. The cell e.s.d.'s are taken into account in the estimation of distances, angles and torsion angles
Refinement. Refinement of *F*^2^ against ALL reflections. The weighted *R*-factor *wR* and goodness of fit *S* are based on *F*^2^, conventional *R*-factors *R* are based on *F*, with *F* set to zero for negative *F*^2^. The threshold expression of *F*^2^ > σ(*F*^2^) is used only for calculating *R*-factors(gt) *etc*. and is not relevant to the choice of reflections for refinement. *R*-factors based on *F*^2^ are statistically about twice as large as those based on *F*, and *R*- factors based on ALL data will be even larger.

### Fractional atomic coordinates and isotropic or equivalent isotropic displacement parameters (Å^2^)

**Table:**

	*x*	*y*	*z*	*U*_iso_*/*U*_eq_	
S1	0.48892 (7)	−0.18020 (5)	0.00175 (3)	0.0512 (2)	
N1	0.5269 (2)	0.13362 (16)	0.09627 (10)	0.0456 (4)	
N2	0.4898 (2)	0.29617 (16)	0.22417 (10)	0.0498 (5)	
N3	0.5397 (2)	0.43946 (17)	0.23930 (11)	0.0502 (5)	
N4	0.6332 (3)	0.39225 (19)	0.09806 (13)	0.0659 (7)	
C1	0.5665 (3)	−0.0136 (2)	−0.04286 (12)	0.0460 (5)	
C2	0.6222 (3)	−0.0303 (3)	−0.13344 (14)	0.0565 (7)	
C3	0.6008 (3)	−0.1767 (3)	−0.16735 (14)	0.0562 (7)	
C4	0.5306 (3)	−0.2720 (2)	−0.10199 (12)	0.0497 (5)	
C5	0.4937 (4)	−0.4350 (3)	−0.11161 (16)	0.0737 (9)	
C6	0.5724 (3)	0.1226 (2)	0.00957 (13)	0.0473 (5)	
C7	0.5491 (3)	0.27297 (19)	0.13780 (12)	0.0432 (5)	
C8	0.6228 (3)	0.4940 (3)	0.16478 (17)	0.0654 (8)	
H2	0.663 (3)	0.048 (2)	−0.1784 (19)	0.0678*	
H3	0.629 (3)	−0.210 (3)	−0.2327 (18)	0.0675*	
H3N	0.515 (3)	0.488 (3)	0.2891 (17)	0.0602*	
H5A	0.52067	−0.48427	−0.05354	0.1103*	
H5B	0.57023	−0.47560	−0.16015	0.1103*	
H5C	0.36648	−0.45026	−0.12734	0.1103*	
H6	0.621 (3)	0.209 (2)	−0.0237 (15)	0.0567*	
H8	0.671 (4)	0.595 (2)	0.1599 (17)	0.0785*	

### Atomic displacement parameters (Å^2^)

**Table:**

	*U*^11^	*U*^22^	*U*^33^	*U*^12^	*U*^13^	*U*^23^
S1	0.0642 (3)	0.0540 (3)	0.0354 (2)	−0.0070 (2)	0.0078 (2)	−0.0047 (2)
N1	0.0516 (8)	0.0465 (7)	0.0386 (7)	0.0015 (7)	−0.0023 (7)	−0.0011 (6)
N2	0.0668 (10)	0.0434 (7)	0.0392 (7)	0.0024 (7)	0.0000 (8)	0.0002 (5)
N3	0.0644 (10)	0.0439 (8)	0.0422 (8)	0.0024 (7)	−0.0020 (8)	−0.0053 (6)
N4	0.0895 (14)	0.0554 (10)	0.0529 (10)	−0.0181 (9)	0.0184 (10)	−0.0072 (8)
C1	0.0475 (9)	0.0518 (10)	0.0388 (8)	−0.0018 (7)	0.0027 (8)	−0.0017 (7)
C2	0.0641 (13)	0.0637 (13)	0.0418 (10)	−0.0064 (9)	0.0110 (9)	0.0012 (9)
C3	0.0605 (12)	0.0706 (13)	0.0376 (9)	0.0014 (10)	0.0098 (9)	−0.0096 (9)
C4	0.0515 (10)	0.0573 (10)	0.0404 (8)	−0.0008 (8)	0.0011 (8)	−0.0092 (7)
C5	0.100 (2)	0.0629 (12)	0.0582 (12)	−0.0099 (13)	0.0055 (13)	−0.0160 (10)
C6	0.0521 (9)	0.0499 (9)	0.0398 (8)	−0.0004 (8)	−0.0003 (8)	0.0008 (7)
C7	0.0475 (9)	0.0431 (8)	0.0391 (8)	0.0031 (7)	−0.0022 (8)	0.0003 (7)
C8	0.0827 (16)	0.0504 (11)	0.0630 (13)	−0.0152 (11)	0.0126 (12)	−0.0079 (10)

### Geometric parameters (Å, °)

**Table:**

S1—C1	1.7169 (19)	C1—C6	1.432 (3)
S1—C4	1.7220 (18)	C2—C3	1.406 (4)
N1—C6	1.286 (2)	C3—C4	1.364 (3)
N1—C7	1.391 (2)	C4—C5	1.490 (3)
N2—N3	1.350 (2)	C2—H2	1.00 (2)
N2—C7	1.324 (2)	C3—H3	1.00 (3)
N3—C8	1.318 (3)	C5—H5A	0.9600
N4—C7	1.355 (3)	C5—H5B	0.9600
N4—C8	1.321 (3)	C5—H5C	0.9600
N3—H3N	0.85 (3)	C6—H6	0.974 (19)
C1—C2	1.365 (3)	C8—H8	0.97 (2)
			
S1···N1	3.1295 (15)	N4···H5A^vii^	2.5700
S1···C4^i^	3.647 (2)	N4···H6	2.39 (2)
N1···S1	3.1295 (15)	C1···N4^v^	3.419 (3)
N1···N3^ii^	2.963 (2)	C4···S1^viii^	3.647 (2)
N2···N4	2.252 (2)	C8···N2^iii^	3.241 (3)
N2···C8^ii^	3.241 (3)	C7···H3^vi^	3.03 (2)
N2···N3^ii^	3.243 (2)	C7···H3N^ii^	2.80 (3)
N3···N1^iii^	2.963 (2)	H2···N2^iv^	2.83 (2)
N3···N4	2.171 (2)	H2···N3^iv^	2.87 (2)
N3···N2^iii^	3.243 (2)	H3···N2^ix^	2.94 (2)
N4···C1^iv^	3.419 (3)	H3···C7^ix^	3.03 (2)
N4···N3	2.171 (2)	H3N···N1^iii^	2.12 (3)
N1···H3N^ii^	2.12 (3)	H3N···N2^iii^	2.77 (3)
N2···H8^ii^	2.71 (2)	H3N···C7^iii^	2.80 (3)
N2···H2^v^	2.83 (2)	H5A···N4^x^	2.5700
N2···H3^vi^	2.94 (2)	H6···N4	2.39 (2)
N2···H3N^ii^	2.77 (3)	H8···N2^iii^	2.71 (2)
N3···H2^v^	2.87 (2)		
			
C1—S1—C4	92.13 (9)	N1—C7—N4	125.46 (16)
C6—N1—C7	116.76 (15)	N2—C7—N4	114.44 (16)
N3—N2—C7	102.19 (14)	N3—C8—N4	110.7 (2)
N2—N3—C8	110.20 (17)	C1—C2—H2	128.6 (14)
C7—N4—C8	102.42 (18)	C3—C2—H2	117.8 (14)
C8—N3—H3N	125.6 (17)	C2—C3—H3	125.2 (15)
N2—N3—H3N	124.1 (17)	C4—C3—H3	121.9 (15)
S1—C1—C6	123.72 (14)	C4—C5—H5A	109.00
C2—C1—C6	125.52 (19)	C4—C5—H5B	109.00
S1—C1—C2	110.75 (16)	C4—C5—H5C	110.00
C1—C2—C3	113.4 (2)	H5A—C5—H5B	109.00
C2—C3—C4	112.85 (18)	H5A—C5—H5C	109.00
S1—C4—C3	110.91 (15)	H5B—C5—H5C	109.00
C3—C4—C5	128.07 (19)	N1—C6—H6	120.2 (12)
S1—C4—C5	121.02 (15)	C1—C6—H6	115.6 (12)
N1—C6—C1	124.19 (17)	N3—C8—H8	124.6 (15)
N1—C7—N2	120.07 (16)	N4—C8—H8	124.7 (15)
			
C1—S1—C4—C3	−0.12 (18)	C8—N4—C7—N2	−0.4 (3)
C4—S1—C1—C2	−0.28 (18)	C7—N4—C8—N3	0.4 (2)
C4—S1—C1—C6	−179.69 (19)	C8—N4—C7—N1	−178.6 (2)
C1—S1—C4—C5	178.8 (2)	C2—C1—C6—N1	−176.6 (2)
C6—N1—C7—N4	−7.9 (3)	S1—C1—C2—C3	0.6 (2)
C7—N1—C6—C1	177.03 (19)	C6—C1—C2—C3	−180.0 (2)
C6—N1—C7—N2	173.91 (18)	S1—C1—C6—N1	2.7 (3)
C7—N2—N3—C8	0.1 (2)	C1—C2—C3—C4	−0.7 (3)
N3—N2—C7—N1	178.54 (17)	C2—C3—C4—S1	0.5 (2)
N3—N2—C7—N4	0.2 (2)	C2—C3—C4—C5	−178.4 (2)
N2—N3—C8—N4	−0.3 (2)		

Symmetry codes: (i) *x*−1/2, −*y*−1/2, −*z*; (ii) −*x*+1, *y*−1/2, −*z*+1/2; (iii) −*x*+1, *y*+1/2, −*z*+1/2; (iv) *x*+1/2, −*y*+1/2, −*z*; (v) *x*−1/2, −*y*+1/2, −*z*; (vi) −*x*+3/2, −*y*, *z*+1/2; (vii) *x*, *y*+1, *z*; (viii) *x*+1/2, −*y*−1/2, −*z*; (ix) −*x*+3/2, −*y*, *z*−1/2; (x) *x*, *y*−1, *z*.

### Hydrogen-bond geometry (Å, °)

**Table:**

*D*—H···*A*	*D*—H	H···*A*	*D*···*A*	*D*—H···*A*
N3—H3n···N1^iii^	0.85 (3)	2.12 (3)	2.963 (2)	172 (2)
C6—H6···N4	0.974 (19)	2.39 (2)	2.761 (3)	101.7 (15)

Symmetry codes: (iii) −*x*+1, *y*+1/2, −*z*+1/2.
